# Pathogenesis of Chicken Astrovirus Related Illnesses

**DOI:** 10.3389/fvets.2022.899901

**Published:** 2022-06-10

**Authors:** Abdullahi Abdullahi Raji, Abdul Rahman Omar

**Affiliations:** ^1^Department of Veterinary Pathology, Faculty of Veterinary Medicine, Usmanu Danfodiyo University Sokoto, Sokoto, Nigeria; ^2^Laboratory of Vaccine and Biomolecules, Institute of Bioscience and Department of Veterinary pathology and Microbiology Universiti Putra Malaysia, Serdang, Malaysia

**Keywords:** chicken astrovirus, pathogenesis, pathology, animal model, genotypes

## Abstract

Of the several known viruses, chicken astrovirus (CAstV) has been associated with diarrhea, runting-stunting syndrome, severe kidney disease, and gout, and white chick syndrome (WCS) in young broiler chicks. Discovered in 2004, CAstV consists of two genogroups with an expanding subgroup because of the diversity exhibited in its viral capsid sequence. Despite these findings, there exists a dearth of knowledge on its pathogenesis. This review highlights the pathogenesis and development of *in vivo* and *in vitro* models.

## Introduction

Chicken astroviruses (CAstVs) are small, round, non-enveloped, positive-sense, single-stranded RNA viruses in the avian astrovirus genus of the *Astroviridae* family. They are ubiquitous in healthy and sick chickens and are capable of causing significant economic losses during infection. The prevalence and incidence of the virus have been reported in the UK, the US, Germany, China, Finland, Poland, Nigeria, South Africa, the Netherlands, and the Middle East (1–7). In the UK and Malaysia, a seroprevalence investigation reveals seropositivity of CAstV in four generations of broilers and broiler breeder flocks, respectively, (8, 9). Since the initial detection of CAstV in the flocks of commercial broiler chickens in 2004 (10), new strains of CAstV have been identified and characterized in chickens worldwide (5, 9, 11–17).

Previously, infections with astroviruses were assumed to be species specific; however, there are pieces of evidence strongly suggesting transmission between the avian species, such as turkey, chicken, and duck astroviruses, while exhibiting some share of identity in their genetic features (1, 18). In 2009, Todd et al. in a seroprevalence study, discovered CAstV antibodies in turkey breeder flocks in the UK. Similarly, a serosurvey on poultry abattoir workers has shown that the antibodies against turkey astrovirus are three times higher in the turkey processing workers when compared to the workers not in contact with turkey (19). Recently, CAstV was detected in cases of runting in day-old commercial turkey poults in southwestern Nigeria (20), and conceivably, this finding evidenced the possibility of cross-species transmission of CAstV to other avian species.

Despite the progress made in the detection and characterization of novel CAstVs (9, 14, 17, 21, 22), there is still a dearth of knowledge about its pathogenesis. Studies on both human and turkey astroviruses have demonstrated an increase in the permeability of intestinal epithelial cells, leading to the disruption of tight junctions between the cells (23, 24). Because the digestive system relies on cellular tight junctions to demarcate the lumen from the basal lamina, a loss of integrity increases water, solute, and ion movements through compartments, limiting the intestine's reabsorption capability and thus resulting in diarrhea (25). Furthermore, extra-intestinal astrovirus-related conditions have been reported in chickens, animals, and humans (11, 12, 26, 27), which could have stemmed from increased intestinal permeability. In addition, astroviruses, even while not replicating, exacerbate the permeability of renal epithelial cells (28). Although the exact pathway leading to diarrhea and spread to other organs/systems is continuously under investigation, the collective findings with turkey astrovirus (TAstV-2) suggest that astrovirus infection causes malabsorption of sodium salts, presumably as a result of redistribution of sodium transporters, thus resulting in osmotic diarrhea (25).

Like the turkey model, the murine model (murine astrovirus, MuAstV) was discovered by researchers in mice in 2012. However, in contrast to TAstV, diarrhea was absent in the infection caused by MuAstV. This finding is expected in mice because colitis must be induced before diarrhea (29, 30). Moreover, no intestinal damage was observed after the MuAstV infection. However, the virus caused an increase in intestinal permeability, indicating that increased permeability in the mouse model could be independent of diarrhea.

Recently, studies in chickens have helped to elucidate the molecular and cellular attributes of CAstV conditions (14, 31). This review, therefore, focuses on some of these findings and tries to outline the critical steps that need to be considered for further research on astroviruses.

## Chicken Astrovirus (CAstV) Associated Diseases

Shortly after the discovery of astrovirus in 1975, small round virus particles with astrovirus morphology were recorded in domestic animals, particularly calves and lambs with gastroenteritis (32, 33) and ducks with viral hepatitis in the early 1980s leading to acute mortality (34), which conceivably served as the earliest indication of extra-intestinal tropism of astrovirus (35). Over the years, chickens, especially the broiler types, are found to be susceptible to varying conditions caused by astrovirus (4, 9). Within a few years of discovery, two distinct genogroups of CAstV (Group-A and Group-B) have been described. Group-B with six subgroups (Bi, Bii, Biii, Biv, Bv, and Bvi) is the most common worldwide and is majorly responsible for the three types of conditions related to CAstV (4, 9), while Group-A with three subgroups (Ai, Aii, and Aiii) is documented to be responsible for two out of the three conditions (4). However, there exists an extensive variation with regards to the geographic distribution in the prevalence of the conditions related to subgroup-B strains, and their link with the three clinical conditions remains unknown. For example, FP3 of the Bi subgroup, initially isolated from a dead-in-shell embryo in the UK, causes intestinal lesions 24 h post-inoculation and severe kidney lesions from day 1 up to 8 days post-inoculation (dpi) in specific pathogen-free (SPF) chickens (4, 36, 37), whereas the Indian strains have been identified to cause severe kidney disease with visceral gout in both broiler and SPF chickens (11). Similarly, with the exception of PL/059/2014 strain of Poland, a member of the Aiii subgroup, all the other strains responsible for white chick syndrome (WCS) originating from Canada and Brazil are clustered in Biv. Recently, in a study using SPF chickens, a Malaysian CAstV strain (UPM1019/2018) caused nephropathy and gouty lesions but were less severe compared to the UK and Indian strains (9).

Clinical manifestations vary from diarrhea (3–4 days after infection), to runting-stunting syndrome (6–12 days post-hatch), to weak and runted chicks in cases of hatchery disease or WCS, and to death, as a result of nephropathy and visceral gout, or in severe cases of WCS (10–12, 14, 31, 38). In a breeder flock, the clinical symptom is characterized by decreased production and hatchability, or mid-to-late dead-in-shell embryo (14, 26, 28, 38).

Chicken astrovirus infections are of clinical and economic concern due to their negative impact on production, owing to the increased severity of clinical symptoms and extra-intestinal involvement. Although other viruses have been implicated in cases of RSS, underperformance, and growth checks, recent studies have identified CAstV as the sole etiologic pathogen responsible for the condition in chickens and turkeys (10, 12, 20, 39). Similarly, DNA and RNA extracted from birds exhibiting underperformance, growth checks, and amplified using various enteric virus-specific primers were consistently positive for CAstV but negative for other enteric viruses (9, 17, 40). Next-generation sequencing and metagenomic approach, as an alternative, have been used to detect the presence of CAstV in the clinical samples (6, 41, 42). An experimental infection study with two US strains (CkP5 and CC_CkAstV) of subgroup-Biv (12) and a Malaysian strain (UPM1019/2018) of subgroup-Bv (9) presented decreased weight gain and cystic lesions in broiler and SPF chicks, respectively. These results are characteristic of RSS.

Furthermore, over the years, severe kidney disease and gout in chickens were associated with infectious bronchitis virus (IBV), avian nephritis virus (ANV) (a serologically and antigenically similar astrovirus identified before CAstV in chickens), and nutritional or managemental factors, but recently, these symptoms have been associated with CAstV (11, 39, 43, 44). Comparatively, the lesions caused by CAstV strains responsible for these conditions are severe and reported to cause 67.5 and 100% mortality in experimentally infected day-old broiler and SPF chickens, respectively. Remarkably, in a study by Bulbule et al. (11), a lower mortality rate was noticed in the broiler chickens when compared to that observed in the SPF chickens. This finding could be attributed to the presence of circulating maternal antibodies against CAstVs in the broiler chickens, which indicates that vaccination of broiler breeders against CAstV could possibly protect the chickens against CAstV infection during the first few days of life, thus preventing the disease.

On the other hand, kidney disease has also been observed in chickens with WCS in addition to the lesions seen on the feathers, intestine, and liver (4, 5, 14, 26, 38). The WCS was first recognized in the mid-1980s in Norway, Finland, Sweden, the US, and Canada (38, 45), with several cases documented in 2016 in Canada, Poland, Brazil, and the US (5, 40, 45, 46). Primarily, the disease is transmitted vertically and mostly affects the broiler-type of chicken (5, 47). A decrease in hatchability of 4 to 68%, mid-to-late dead-in-shell embryo and 100% mortality within the first few hours of weak hatched chicks have been documented in WCS (38, 46). The chickens affected by WCS present an enlarged intestine filled with yellow-green fluid and air bubbles, thus leading to decreased nutrient absorption by the intestinal epithelium (14, 25, 31, 48). The microscopic changes (biliary proliferation, accumulation of immature granulocytes, hyperplasia, and dilated epithelium with heterophils and eosinophilic debris) are observed in the liver and bile duct but absent in the intestines. The absence of microscopic lesions in the intestines was recently attributed to the activation of immunosuppressive cytokine, transforming growth factor-beta (TGF-β), during the course of CAstV infection (31). In addition, these alterations could lead to a lack of consumption and absorption of the yolk by the intestinal epithelium within the first few days of embryogenesis. The yolk sac is known to provide nourishment and equally supports the embryo in its early stage of life, and provides carotenoids for the maintenance of body movements before a normal yellow chick is hatched. This is primarily achieved by the utilization of zeaxanthin and lutein from the yolk sac (49, 50). WCS-affected chickens have a bigger and heavier yolk sac, as well as lower yolk absorption, leading to weak and uncolored chicks (31). Although the cause of depigmentation in “white chicks” is not known, it is speculated that alterations in the synthesis of melanin during embryogenesis could be responsible for this condition (40, 45, 46, 51). This alteration equally affects the eyes, as observed in chickens huddling together in a well thermoregulated pen house (52).

There are numerous similarities between the astrovirus infections in chickens and other animal species, including humans. Most animals infected with astrovirus, including humans, calves, piglets, turkeys, and lambs, develop diarrhea similar to chickens (4, 32, 53–57). However, some animal species remain asymptomatic (23, 33, 58). Therefore, the epidemiology and transmission mechanisms leading to asymptomatic astroviral infection in chickens and other animals, as corroborated by Schultz-Cherry et al. (22), remain unclear. Advancement in the area of molecular techniques will contribute to a greater understanding of the varying conditions of CAstV and other animal-related astroviral infections in terms of prevalence and genotype-specific infections.

## Cell Culture And Animal Model

As with other astroviruses, the pathogenicity of CAstV remains unknown, although factors like chicken type and host age, virus strain and concentration, co-infections with other viruses, and maternal antibodies could influence the disease pattern (4). Similarly, propagation and isolation of the CAstV and other astroviruses in an *in vitro* system, such as embryonated chicken egg or cell culture, is complex and unpredictable (12, 59). Nevertheless, CAstV strains can replicate in different cell lines, and liver-derived LMH, chicken embryo kidney cells (CEKC), and chicken embryo liver (CEL) cell line are frequently used (10–12, 20, 39). LMH seems to be a suitable, permissive, and susceptible host cell line for the propagation and isolation of CAstV. In addition, different sites and routes in 5- to 8-day-old embryonated SPF chicken eggs, including chorioallantoic membrane (CAM) (5) and yolk sac (40, 60–62), have also been employed to isolate CAstV. However, some cell lines, such as MDCK, DFI, QM7, Vero, and Sf9, did not support the propagation of the virus (12).

The non-canonical HAstV-MLB (Melbourne) (MB1 to 3) and HAstV-VA/HMO (Virginia/Human-Mink-Ovine-like) strains were not supported by any cell line for propagation, similar to the majority of other avian and mammalian astroviruses (14, 62). However, with the identification of the human intestinal enteroids (HIE) (59) in 2020, the propagation of these viruses became possible. The reason behind why some isolates developed while others failed to grow is yet to be established. Unfortunately, till the receptor(s) of astrovirus are known, creating an appropriate cell culture system will be difficult (22, 58). With several groups working on the identification of astrovirus biding sites, crystallization of human and turkey astroviruses has discovered a polysaccharide motif in the capsid and spike proteins (22, 63, 64). As described by Kang et al. (12), trypsin usage in isolating CAstV in LMH cell line is not required as indicated for bovine, human, and swine astroviruses (59, 65–67).

In Kang et al. (12) study, cytopathic effect (CPE) and the detachment of predominant small round cells were observed in the third passage 72 and 120 h post-inoculation (pi), respectively, after inoculation of the subsequent passage. The observed CPE was further confirmed to be as a result of CAstV by using the alpha-beta neutralization test (12). Furthermore, the replication kinetics of CAstV was observed to be associated with the cell at 1 h pi with a few supernatant virions. Twelve hours later, the viral eclipse was noticed, with a complete replication cycle at 12 and 24 h. Analyses of post-inoculation time points (24, 48, 72, and 96 h) revealed a constant increase in the virus titers in both supernatant and cells (12).

Fluorescent *in situ* hybridization (FISH) of CAstV suggests that the replication of the virus is restricted to the enteric enterocytes. In the epithelial cells of the villous, the identification of the viral RNA was explicit within the first 12 hpi (12). The site of CAstV staining shifted from the villous epithelial cells to the crypt epithelial cells at 18 h pi. Viral RNA was detected abundantly in the crypt epithelial cells and also in the attenuated epithelial cells in a dilated crypt within 48 hpi (12).

In an experimental infection study using SPF day-old chickens, individual degenerating enterocytes of the small intestines were apparent along the basal edge of the villi by 3 dpi and continued through 5 dpi at the crypt epithelial cells. By 5 dpi, CAstV localized primarily in the small intestine. By 6 dpi, mild shortening of the villi was prominent, as well as the occasional clusters of necrotic enterocytes along the villous base correlating with the infection (22). Replication peaked at 2–5 dpi, and CAstV was intermittently observed at time points later than 12 dpi (12). Although CAstV restricts its viral RNA replication to the small intestine, its isolation from the spleen, kidney, thymus, bursa of Fabricius, pancreas, gut content, and liver in varying quantities, via conventional and real-time PCR, shows that it can be detected in a wide range of tissues (1, 14, 31, 61). Based on the replication kinetics of CAstV, the findings of the study indicated that villous epithelial cells were initially permissive and susceptible to CAstV infection but later became refractory to the entry of the virus. However, the virus disseminated and multiplied in the crypts of Lieberkühn. Since crypt epithelial cells actively divide and migrate toward the villi, it is hypothesized that CAstV would prefer immature intestinal cells. In addition, the CAstV replication cycle is documented to be short and self-limiting within a few days of infection (12, 68, 69).

Furthermore, immunity-related studies of CAstV showed that the relative levels of gene expression of some of the important cytokines are different in the various tissue samples, which include thymus, jejunum, liver, and spleen, of the chickens infected with CAstV-WCS isolates (31). It was also observed that several T-helper cells (Th1 and 2) were activated in response to these cytokines. While IFN-γ, IL-2, IL-8, IL-12 p40, IL-15, TGF-β4, TNF-SF-15, and t-BET were expressed in the liver of the chicks, some of the cytokines were expressed in the thymus, spleen, and jejunum. Interestingly, even with the significant viral concentrations in the spleen and jejunum, there was a lack of IFN-γ activation in both organs (31). At the nexus of non-specific and acquired immunities, increased IFN-γ expression, as well as the activation of IFN-γ-secreting NK and Th1 cells, indicates that the innate immune response is equally triggered (31). Similar to the turkey model (48), lesions were found to be absent in the gut epithelium due to the activation of transforming growth factor-beta (TGF-β), a potent immunosuppressive cytokine which was significantly expressed in the jejunum of the chickens infected with the CAstV-WCS isolates, thus leading to the downregulation of inflammatory response and consequently, absence of microscopic lesions (31, 70).

Furthermore, in Nuñez et al. (31) study, the liver expressed the highest levels of IL-8 and IL-15. In the same vein, a considerable quantity of heterophiles and lymphocytes were observed to infiltrate the liver. It is known that, in cases of viral infections, IL-15 exerts an anti-apoptotic effect on the cytotoxic cells, while keeping them alive, and simultaneously destroys a large number of infected cells (31). In contrast, in the liver, IL-8 contributes to the infiltration of heterophiles and macrophages (31). Moreover, in CAstV-WCS-related infections, TNF-SF-15 and IL-12 p40 were also observed in higher concentrations in the liver. As a cytokine expressed by endothelial cells, TNF-SF-15 induces apoptosis and reduces proliferation in an autocrine manner, which potentially explains the observed microscopic apoptotic processes. Accordingly, the activation of innate immunity is initiated by the expression of IL-12 p40 in an attempt to establish cellular-mediated immunity *via* Th1-mediated response.

With all these fascinating and limited findings and works on the pathogenesis of CAstV, it will be quite interesting to study the virus further.

## Conclusion

With much still needed to be worked on, particularly on the pathogenesis of the virus, there has been progress in the areas of molecular characterization and typing of the virus. The recent discovery of Bv and Bvi subgroups reveals that CAstV-related conditions are widespread and affect the commercial poultry industry to a much greater extent than previously anticipated (9). In the same vein, the emergence of these novel CAstVs clearly shows the possibility of the virus to spread systematically and cause varying pathologies in chickens, as observed in human and bovine astroviruses that have been reported to cause meningitis and encephalitis. Similar to other astroviruses, it has also been established that CAstV causes diarrhea in the absence of microscopic lesions, by disrupting tight junctions and allowing water influx, and finally diarrhea. In addition, studies in the turkey and murine models revealed that the viral capsid alone can cause diarrhea *in vivo*. Even with all these advances in the study of CAstV and other astroviruses, we, nonetheless, confront hurdles in having a standardized animal model that will elucidate the pathogenesis of astrovirus and the subsequent production of the vaccine, therapeutics, and cross-species infections. Unlike HAstV, chickens or turkey poults can perfectly be utilized for the studies related to CAstV pathogenesis and hence serve as a basis for further research on astrovirus.

## Author Contributions

AR: conceptualization, writing-preparation and writing of the first draft, writing-review and editing. AO: concept, formatting, initial review, approval for publication and agree with final publication of the article.

## Conflict of Interest

The authors declare that the research was conducted in the absence of any commercial or financial relationships that could be construed as a potential conflict of interest.

## Publisher's Note

All claims expressed in this article are solely those of the authors and do not necessarily represent those of their affiliated organizations, or those of the publisher, the editors and the reviewers. Any product that may be evaluated in this article, or claim that may be made by its manufacturer, is not guaranteed or endorsed by the publisher.
